# Brer Rabbit and the Tar Baby

**Published:** 1900-06

**Authors:** 


### EDITORIAL NOTES AND COMMENTS.

The Business office of THE JOURNAL-RECORD is 305, 306 Fitten Building.

The Editorial office is 318-319-320 The Prudential.

Address all Business communications to Dr. M. B. Hutchins, Mgr.

Make remittances payable to THE ATLANTA JOURNAL-RECORD OF MEDICINE.

On matters pertaining to the Editorial and Original Communications address Dr. Bernard Wolff, Atlanta.

Reprints of original articles will be furnished at cost price. Requests for the same should always be made *on the manuscript*.

We will present, post-paid, on request, to each contributor of an original article, twenty (20) marked copies of THE JOURNAL-RECORD containing such article.

### BRER RABBIT AND THE TAR BABY.

SOME of our esteemed advertising patrons seem exercised over the rumor of a contemplated debauch of the materia medica section of the American Medical Association by various farben fabriken Gesellschaften and coal-tar paranoiacs from overseas. Has it really come to the pinch? Shall we stand by and witness with unruffled indifference the rape of our nurtured industries? Will the friends of American enterprise permit the eagle bird of freedom to be plucked like a hissing goose and its devoted plumage go to stuff the pillow upon which rests the cotton-plugged ear of the Teutonic competitor? All our national pride, interest and patriotism join in the swelling chorus, "Nit!"

It is a little dim to our comprehension what the foreign chemical houses expect to accomplish by "fixing" the materia medica section. This is not the crystallized unit of the medical profession in this country, nor would its absence throw a very heavy strain on Christian fortitude to endure. We were not aware that the decisions of this section influenced the course and destiny of

American pharmaceutical products, or, indeed, that it influenced anything or anybody. The only influence apparent is that which might be exercised upon the ineffaceable instinct of simian mimicry that leads men to imitate other men and to follow blindly the suggestions of those whom, in point of professional acumen, they regard as above them. But this is not sufficient to alter the prepossession of those who have taken fast hold of instruction, and whose faith in drugs whose uses they understand and whose value they appreciate is not to be shaken by the specious allurements of a passing fad.

There is no prejudice here against imported therapeutic products. In fact, they are generally regarded with favor. It seems to us it would be indiscrete for foreign chemical houses to jeopardize the friendly feeling toward their wares by attempting to exploit them by chicanery, or to run out of the sphere of popular use and estimation articles of domestic manufacture by a *tour de force*. Such policy would be at cross purposes with a fine sense of patriotism in their patrons, which is not apparent unless the occasion develops it, when it is apt to mature into absolutely annihilating public opinion.

No, Brer Rabbit is not yet to be caught by the Tar Baby.

---

A PAPER was presented at the recent meeting of the State Medical Association by Dr. Henry R. Slack, of LaGrange, which urged the establishment of a Pasteur Institute in Georgia for the treatment of hydrophobia. The writer had collected from various sources seventy or more cases of rabies occurring in this State. A few of these unfortunates had been sent to the Pasteur establishment conducted by Dr. Gibier in New York; but the remainder, whose death was not chronicled in the secular press, had disappeared from view, having impliedly met a melancholy fate.

The suggestions contained in the paper were adjudicated upon by a committee especially appointed, who reported favorably, and a Pasteur Institute in Atlanta is now to be contemplated as a development in the near future.

Without entering into a discussion of the vexed question of hydrophobia, a disease whose actual existence is strenuously denied by many whose opinions are entitled to respect, it seems to us that there is a more apparent need for hospitals and similar establishments of larger design and wider field of usefulness than one of such avowed restriction as the Pasteur Institute. There is no institution in this city or elsewhere in the State, so far as we are aware, devoted to the care and treatment of the incurably sick who are unable to maintain themselves and who have no one to support them. There is no place for the numerous sufferers from chronic disease of the organs essential to life in the city hospital. Such afflicted individuals, if in indigent circumstances, inevitably become wards of a meager, reluctant and ungracious charity, or perish miserably and alone.

The writer recalls the case of a wretched negress, bedridden, helpless and dying from an immense and destructive cancer of the hip, without sufficient strength to keep the greedy, persistent and horrible flies from their repulsive feast. Her sole and occasional attendant was a young mulatto strumpet, who sustained her from her slender and equivocal earnings.

Where there are seventy cases of hydrophobia in the medical annals of the State, there are in our midst ten times that number of such instances as that cited of dismantled and abandoned derelicts drifting to the port of missing ships. In the interest of public good there could be no more fitting disposal of municipal charity than in founding and maintaining a hospital for incurables.

There is, further, in this city no establishment for the isolation and treatment of cases of contagious disease other than smallpox, and the assumption is justifiable that much of the spread of communicable disease in children, especially of the lower classes, proceeds from necessarily defective and insufficient household isolation practiced for lack of means of segregation.

House quarantine, as it exists in this city, is a transparent but menacing farce.

The hospital facilities which we already have are taxed to their utmost to accommodate the demand made upon them. The Grady Hospital is inadequate in space to house the patients entitled to admission. This is especially true of the negro wards, which are always crowded, forcing patients needing hospital attention to wait until death or convalescence supplies an empty bed.

It is desired that these remarks shall not be construed as being adverse to the establishment of a Pasteur Institute. We hail progress and advance in medical matters with unalloyed satisfaction; but it is more gratifying when such progress is concerned with a need that is daily and constant rather than one which is fluctuating and occasional.

---

DR. HOWARD J. WILLIAMS of Macon, Ga., an ex-president of the Georgia Medical Association, who has been in general practice and surgery for the last eighteen years, will, on the 1st of June, limit his work entirely to general surgery. The firm of McHatton & Williams will at that time be dissolved, Dr. McHatton continuing his general practice, and Dr. Williams limiting himself as above stated. Both gentlemen are well known throughout the State.

